# The Isolation and Characterization of Rare Mycobiome Associated With Spacecraft Assembly Cleanrooms

**DOI:** 10.3389/fmicb.2022.777133

**Published:** 2022-04-26

**Authors:** Adriana Blachowicz, Snehit Mhatre, Nitin Kumar Singh, Jason M. Wood, Ceth W. Parker, Cynthia Ly, Daniel Butler, Christopher E. Mason, Kasthuri Venkateswaran

**Affiliations:** ^1^Biotechnology and Planetary Protection Group, Jet Propulsion Laboratory, California Institute of Technology, Pasadena, CA, United States; ^2^Department of Physiology and Biophysics, Weill Cornell Medicine, New York, NY, United States; ^3^The WorldQuant Initiative for Quantitative Prediction, Weill Cornell Medicine, New York, NY, United States

**Keywords:** mycobiome, cleanroom, chloramphenicol, metagenome, amplicon sequencing

## Abstract

Ensuring biological cleanliness while assembling and launching spacecraft is critical for robotic exploration of the solar system. To date, when preventing forward contamination of other celestial bodies, NASA Planetary Protection policies have focused on endospore-forming bacteria while fungi were neglected. In this study, for the first time the mycobiome of two spacecraft assembly facilities at Jet Propulsion Laboratory (JPL) and Kennedy Space Center (KSC) was assessed using both cultivation and sequencing techniques. To facilitate enumeration of viable fungal populations and downstream molecular analyses, collected samples were first treated with chloramphenicol for 24 h and then with propidium monoazide (PMA). Among cultivable fungi, 28 distinct species were observed, 16 at JPL and 16 at KSC facilities, while 13 isolates were potentially novel species. Only four isolated species *Aureobasidium melanogenum*, *Penicillium fuscoglaucum*, *Penicillium decumbens*, and *Zalaria obscura* were present in both cleanroom facilities, which suggests that mycobiomes differ significantly between distant locations. To better visualize the biogeography of all isolated strains the network analysis was undertaken and confirmed higher abundance of *Malassezia globosa* and *Cyberlindnera jadinii*. When amplicon sequencing was performed, JPL-SAF and KSC-PHSF showed differing mycobiomes. Metagenomic fungal reads were dominated by Ascomycota (91%) and Basidiomycota (7.15%). Similar to amplicon sequencing, the number of fungal reads changed following antibiotic treatment in both cleanrooms; however, the opposite trends were observed. Alas, treatment with the antibiotic did not allow for definitive ascribing changes observed in fungal populations between treated and untreated samples in both cleanrooms. Rather, these substantial differences in fungal abundance might be attributed to several factors, including the geographical location, climate and the in-house cleaning procedures used to maintain the cleanrooms. This study is a first step in characterizing cultivable and viable fungal populations in cleanrooms to assess fungal potential as biocontaminants during interplanetary explorations. The outcomes of this and future studies could be implemented in other cleanrooms that require to reduce microbial burden, like intensive care units, operating rooms, or cleanrooms in the semiconducting and pharmaceutical industries.

## Introduction

The primary aim of the National Aeronautical Space Administration (NASA) Planetary Protection (PP) program is to prevent forward and back contamination between Earth and other celestial bodies (Rummel et al., 2002). To comply with these requirements, rigorous maintenance procedures have been implemented in the cleanrooms where spacecrafts are assembled, including the spacecraft assembly facility (SAF) at Jet Propulsion Laboratory (JPL) and Payload Hazardous Servicing Facility (PHSF) at Kennedy Space Center (KSC) (La Duc et al., 2003, 2004). Nevertheless, despite stringent “housekeeping” rules, reports have shown that viable microbial populations can be retrieved (La Duc et al., 2004, 2012; Weinmaier et al., 2015).

Humans may be considered walking incubators for microorganisms (Meadow et al., 2015), shedding commensal microorganisms to any environment they inhabit. These cast-off microorganisms influence persisting populations in closed habitats, including Lunar/Mars Analog Habitat (ILMAH) (Mayer et al., 2016; Blachowicz et al., 2017), Mars500 facility (Mora et al., 2016) and SAF cleanroom (Weinmaier et al., 2015). Since bacteria are predominant in the human (Meadow et al., 2015; Sender et al., 2016) closed habitats’ (La Duc et al., 2003, 2004, 2012), ILMAH (Mayer et al., 2016), Mars500 (Mora et al., 2016) and the International Space Station (ISS) microbiomes (Checinska et al., 2015; Singh et al., 2018; Checinska Sielaff et al., 2019), the majority of conducted environmental studies have focused on investigating their resilience and pathogenicity (Bashir et al., 2016; Singh et al., 2018; Urbaniak et al., 2018). However, despite lower abundance in JPL cleanrooms (∼2–3% of all isolated and identified microorganisms) (Hendrickson et al., 2017), fungi should not be disregarded in the context of future outer space exploration. Fungal survival in space environments can be fostered by their hardy nature and capacity to produce a myriad of bioactive compounds (Blachowicz et al., 2019a; Keller, 2019).

Enormous phenotypic plasticity and adaptability allow fungi to persist and grow in extreme (Zhdanova et al., 2004; Sterflinger et al., 2012) and built environments (Adams et al., 2013; Blachowicz et al., 2017). Traditional assays employed by NASA PP program target specifically the detection of endospore-forming bacteria (Benardini et al., 2014). Their extreme hardiness allows for tolerating inhospitable conditions for long periods of time, which makes them suitable candidates for surviving the journey to other planetary bodies. However, while fungal species also produce protective structures (spores, conidia, or cysts), often in response to environmental stressors, few studies have examined their survival under simulated space conditions (Gorbushina, 2003; Blachowicz et al., 2019a). A few reports discussed the fungal distribution in assembly facilities (Herring et al., 1974; La Duc et al., 2012), but no systematic characterization utilizing state-of-the-art molecular techniques has been attempted. Since the presence of fungi has been documented in JPL cleanrooms (La Duc et al., 2012; Weinmaier et al., 2015), there is an unmet need to study the complexity of fungal population and its fluctuation over time. Furthermore, in light of emerging research, such analyses are imperative to discern the potential for fungi to be contamination agents while exploring space (Knox et al., 2016; Romsdahl et al., 2018a; Blachowicz et al., 2019b).

Several studies have tested using antibiotics to reduce bacterial populations in environmental or food samples to facilitate the estimation of the fungal components (Cooke, 1954; Koburger and Rodgers, 1978; Tournas et al., 2020). The impact of antibiotics on the growth of selected fungi was tested, revealing that chloramphenicol and gentamicin were effective in reducing bacterial populations without impairing fungal growth (Dolan, 1971). In principle, chloramphenicol reversibly binds to the bacterial 50S ribosomal subunit and interferes with protein synthesis and cell proliferation (Oong and Tadi, 2021). Given that, unlike gentamicin, chloramphenicol is bacteriostatic to both Gram-positive and Gram-negative bacteria, short incubations with it were expected to enhance retrieval of fungi from SAF samples.

This study is the first report focusing mainly on the mycobiomes of two NASA SAF cleanrooms implementing an approach not undertaken before. Collected environmental samples were treated with chloramphenicol for 24 h to suppress bacterial activity. Subsequently, samples were treated with propidium monoazide (PMA) to intercalate DNA released from *dead/damaged* microbial cells. As a result of these treatments, characterization of *viable/intact* fungal populations was possible. Culture- and molecular-based techniques were implemented to profile the composition of SAF mycobiomes. Additionally, fungal diversity was determined by both amplicon and metagenome sequencing to analyze identified communities in terms of their taxonomy, virulence and stress-related functional properties.

## Materials and Methods

### Sampling Locations and Environmental Conditions

Two geographically distinct NASA cleanrooms, JPL-SAF and the KSC-PHSF, were sampled between April and September of 2018. The specific sampling locations and the corresponding environmental data are given in Table 1 and Supplementary Figure 1. Both facilities were maintained with cleaning regimens appropriate to the current level of on-going activities. During samplings at JPL-SAF, assembly activities of critical spacecraft hardware were happening; therefore, SAF was cleaned daily, including vacuuming and mopping with a cleaning solution (Kleenol 30, a highly concentrated, industrial strength heavy-duty cleaner/degreaser, which contains12.5% ethylene glycol monobutyl ether, 1–5% nonylphenol ethoxylate, 1% dodecylbenzenesulfonate, and 1–4% silicic acid, disodium salt). When, KSC-PHSF was sampled, no assembly activity or spacecraft hardware were present, thus daily cleaning included vacuuming and mopping with plain water. Additionally, daily cleaning regimens in both cleanrooms include replacing tacky mats. All personnel who enter these cleanrooms must follow good manufacturing practice procedures to minimize the influx of particulates. Specific entry procedures depend on the certification level of the cleanroom and the presence of mission hardware. General precautions include donning of cleanroom-certified garments to minimize exposure of skin, hair and the regular clothing of personnel. In addition, general precautions prohibit the use of cosmetics, fragrances, body spray and hair gels before entry into the cleanroom. Lastly, the air of both facilities was filtered through high-efficiency particle air filters.

**TABLE 1:** Metadata of samples collected from JPL-SAF and KSC-PHSF.

Facility/Location/ Dimensions [m]	Characteristics^a^	Significance	Sample ID	Abbrev. name	Description	Date	Sampling method	Sampling area	Extraction volume (ml)
Jet Propulsion Laboratory Spacecraft Assembly Facility (JPL-SAF) Pasadena, CA West coast; dry desert like	ISO 7 (Class 10,000) HEPA Room Temperature: 20.4 ± 0.3^°^C Room Humidity: 45.5 ± 2%	Pathfinder (1997); Mars Exploration Rovers (2004); Mars Science Laboratory (2012); Perseverance (2020)	FD-01-001	L1	SW, near desk area and walkway to desk area	4/17/2018	BiSKit	1 m^2^	45
			FD-01-002	L2	SW, corner between desk and front of unused door				
			FD-01-003	L3	SW, desk area & walking area to Cruise Stage				
			FD-01-004	L4	S, between cruise stage and Descent stage				
			FD-01-005	L5	Center, between desk and Descent stage				
			FD-01-006	L6	Center, walking area to desk behind monitor				
			FD-01-007	L7	W Center, next to center walkway, crane, and desk				
			FD-01-008	L8	SE, area in front of high bay spacecraft exit doors				
25 × 36 × 15			FD-01-009	L9	Center, next to storage boxes in center of room				
			FD-01-010	L10	E, directly in front of room entrance, after tacky mat				
			FD-01-HC-01	HC	Handling control (exposed to facility air)			—	
			FD-01-DC-01	DC	Device control (moistened BiSKit, not used)			—	
Kennedy Space Center Payload Hazardous Servicing Facility (KSC-PHSF) Cape Canaveral, FL East coast; swamp-like	ISO 7 (Class 10,000) HEPA Room Temperature: 21.7 ± 3.3^°^C Room Humidity: = 55%	Mars Exploration Rovers (2004); MarsReconn-AissanceOrbiter (2006)Mars Science Laboratory (2012); Perseverance (2020)	FD-03-001	L1	SW, near airlock door for equipment storage moving	6/12/2018	BiSKit	1 m^2^	45
			FD-03-002	L2	S center, between airlock entrance and exit into cleanroom				
			FD-03-003	L3	SE, near wall of high bay, grounding plate included				
			FD-03-004	L4	E center, left of grounding plate				
			FD-03-005	L5	Center, high bay center right				
			FD-03-006	L6	Center, high bay center left				
			FD-03-007	L7	W center, near west wall by fuel drain				
			FD-03-008	L8	NW, corner near west exit door 19 to outside				
33 × 18 × 29			FD-03-009	L9	N center, front of door 18				
			FD-03-010	L10	NE, front of door 17				
			FD-03-HC-01	HC	Handling control (exposed to facility air)			—	
			FD-03-DC-01	DC	Device control (moistened BiSKit, not used)			—	
			FD-03-CM-01	C1	SW, alongside length of grate		ClipperMop	10 m^2^	200
			FD-03-CM-02	C2	Center, high bay center				
			FD-03-CM-03	C3	NE, between door 18 and 17				
			FD-03-CM-HC	CHC	Handling control (exposed to facility air)			—	
			FD-03-CM-DC	CDC	Device control (moistened CM wipe, not used)			—	
Kennedy Space Center Payload Hazardous Servicing Facility (KSC-PHSF) Cape Canaveral, FL East coast; swamp-like	ISO 7 (Class 10,000) HEPA Room Temperature: 21.7 ± 3.3^°^C Room Humidity: = 56%	Mars Exploration Rovers (2004); MarsReconn-AissanceOrbiter (2006)Mars Science Laboratory (2012); Perseverance (2020)	FD-04-001	L1	SW, near airlock door for equipment storage moving	7/24/2018	BiSKit	1 m^2^	45
			FD-04-002	L2	S center, between airlock entrance and exit into cleanroom				
			FD-04-003	L3	SE, near wall of high bay, grounding plate included				
			FD-04-004	L4	E center, left of grounding plate				
			FD-04-005	L5	Center, high bay center right				
			FD-04-006	L6	Center, high bay center left				
			FD-04-007	L7	W center, near west wall by fuel drain				
			FD-04-008	L8	NW, corner near west exit door 19 to outside				
33 × 18 × 29			FD-04-009	L9	N center, front of door 18				
			FD-04-010	L10	NE, front of door 17				
			FD-04-HC-01	HC	Handling Control (exposed to facility air)			—	
			FD-04-DC-01	DC	Device control (moistened BiSKit, not used)			—	
			FD-04-CM-01	C1	SW, alongside length of grate		ClipperMop	10 m^2^	200
			FD-04-CM-02	C2	Center, high bay center				
			FD-04-CM-03	C3	NE, between door 18 and 17				
			FD-04-CM-HC	CHC	Handling control (exposed to facility air)			—	
			FD-04-CM-DC	CDC	Device control (moistened CM wipe, not used)			—	
Jet Propulsion Laboratory Spacecraft Assembly Facility (JPL-SAF) Pasadena, CA West coast; dry desert like	ISO 7 (Class 10,000) HEPA Room Temperature: 20.3 ± 0^°^C Room Humidity: 45.8 ± 0.1%	Pathfinder (1997); Mars Exploration Rovers (2004); Mars Science Laboratory (2012); Perseverance (2020)	FD-05-010	L1	SW, near desk area and walkway to desk area	9/25/2018	Wipe	1 m^2^	45
			FD-05-005	L7	W Center, next to center walkway, crane, and desk				
			FD-05-003	L9	Center, next to storage boxes in center of room				
			FD-05-001	L10	E, directly in front of room entrance, after tacky mat				
			FD-05-002	L11	NE, desk area near entrance				
			FD-05-004	L12	SE, directly front of door to other airlock (unused)				
			FD-05-006	L13	N center, in front of cabinets				
			FD-05-007	L14	Center, between wheels of lift				
			FD-05-008	L15	NW, between GSE				
25× 36 × 15			FD-05-009	L16	NW, between tool boxes				
			FD-05-HC-01	HC	Handling control (exposed to facility air)			—	
			FD-05-DC-01	DC	Device control (moistened wipe not used)			—	
			FD-05-CM-01	C1	E center, near entrance in front of solar panels		Wipe	10 m^2^	200
			FD-05-CM-02	C2	Center, near walkway				
			FD-05-CM-03	C3	E, alongside high bay spacecraft exit doors				
			FD-05-CM-HC	CHC	Handling control (exposed to facility air)			—	
			FD-05-CM-DC	CDC	Device control (moistened CM wipe, not used)			—	

*^a^Characteristic of the cleanroom ISO level is a certification defined by the maximum number of particles of the size >0.5 μm in one cubic foot of air. The cleanrooms’ temperature, humidity, and filter (HEPA, high-efficiency particle filter) are given as its average with± consideration to its min/maximum.*

### Sampling Procedure and Initial Sample Processing

A schematic overview of the methodology implemented to carry out this study is presented in Supplementary Figure 2. Cleanroom floor samplings were performed using three sampling devices: (i) BiSKit; (ii) ClipperMop and (iii) polyester wipe, each of which were premoistened with sterile molecular grade water. The first sampling at JPL-SAF on April 17, 2018 was carried out with BiSKits (Quicksilver Analytics Inc., Abingdon, MD) prepared following the previously described procedure (Blachowicz et al., 2017). Each BiSKit was used to sample a 1 m^2^ area from 10 selected locations (Supplementary Figure 1). Due to the presence of the sensitive payload in JPL-SAF the sampling on September 25, 2018 required changes in the sampling locations and procedure, as the BiSKit is not a flight certified sampling device. Likewise, six sampled locations (L11–L16), where adjusted during the second sampling. Therefore, 10 samples (1 m^2^ each) were collected with 12” × 12” premoistened, flight-certified polyester wipes (Sterile TexTra10 TX3225; Texwipe, Kernersville, NC) and additional three samples (10 m^2^ each) were collected with ClipperMop (TexWipe) using 12” × 12” premoistened, flight certified polyester wipes attached to the mop. Both sampling events at KSC-PHSF on June 12, 2018 and July 17, 2018 were conducted using BiSKits and ClipperMops, collecting samples from 10 to 3 different locations, respectively.

After sampling, BiSKits and corresponding handling and device controls were processed following previously described steps (Blachowicz et al., 2017). Briefly, each sampled BiSKit was extracted three times with 15 mL phosphate buffer saline (PBS) (MoBio Laboratories Inc., Carlsbad, CA) amounting to 45 mL. Polyester and ClipperMop wipes were placed in 500 mL bottle containing 200 mL of sterile PBS, vigorously shaken for 1 min to dislodge microbial cells. These environmental samples were then concentrated using CP-150 InnovaPrep concentrating pipette (Innova Prep LLC, Drexel, MO) to a final volume of ∼6 mL (Kwan et al., 2011). Concentrated BiSKit, polyester wipe and ClipperMop samples were divided into two aliquots (∼2 mL each).

### Chloramphenicol Treatment

Chloramphenicol is a bacteriostatic antibiotic (Oong and Tadi, 2021) that most fungal species resist; therefore, it can be used to suppress bacterial proliferation allowing for isolation of fungi. Aliquots of the concentrated samples were treated with chloramphenicol at final concentration 100 μg/mL and incubated overnight at 25°C along with untreated counterparts. After 18–24 h of incubation, both chloramphenicol treated and untreated samples were processed for various analyses as detailed below.

### Sample Treatment and Assays

#### Cultivable Fungal Burden and Diversity

To assess cultivable burden, samples collected during the first sampling at JPL-SAF (JPL-1) were 10-fold diluted and 100 μL was added in duplicate to potato dextrose agar (PDA) containing chloramphenicol (25 mg/L) and dichloran rose bengal chloramphenicol agar (DRBC) and grown at room temperature (RT) (∼25°C). After 7 days, colony-forming units (CFUs) were enumerated and used to calculate the cultivable fungal population per meter square. All colonies that grew were collected and stored as stab cultures and in glycerol stocks. Based on the observed results for JPL-1, the processing method for the following three sampling events (JPL-2, KSC-1, and KSC-2) was altered as follows. Upon concentration, samples were divided into two equal aliquots, with one treated with chloramphenicol (100 μg/mL) for 24 h while the other was left untreated. Antibiotic-treated and untreated samples were then 10-fold diluted and 100 μL was plated in duplicates on PDA and DRBC containing chloramphenicol. After 7 days of incubation at room temperature, colonies were counted and collected for identification with the internal transcribed spacers (ITS) region using primers ITS 1F (5′-CTT GGT CAT TTA GAG GAA GTA A-3′) (Lai et al., 2007) and Tw13 (5′-GGT CCG TGT TTC AAG ACG-3′) (Taylor and Bruns, 1999). DNA was extracted using the Maxwell-16 MDx automated system following the manufacturer’s instructions (Promega, Madison, WI). PCR conditions and sample preparation steps for sequencing were exactly as described elsewhere (Blachowicz et al., 2017). Sanger sequencing was performed and ITS sequences were identified through the Basic Local Alignment Search Tool (BLAST) algorithm (Altschul et al., 1990) using the National Center for Biotechnology Information (NCBI) database to find the type strains with the closest percent similarity to each isolate.

#### Assessment of UV-C Resistance

To evaluate ultraviolet radiation with wavelengths between 200 and 290 nm (UV-C) sensitivity, approximately 10^2^ conidia/plate were exposed to UV-C. Conidia were suspended in PDA top agar (PDA, containing half of the agar concentration, 7.5 g/L) and 5 mL of this mix was added to plates containing 20 mL of PDA (total agar amount, 15 g/L). Duplicates of each isolate were exposed to 1,000 J/m^2^ using a low-pressure handheld mercury arc UV lamp (model UVG-11; UVP Inc., Upland, CA). Following exposure, treated and untreated (control) plates were incubated at room temperature. After 7 days, colonies were enumerated and relative survival was assessed and calculated using the following formula: no. of CFU exposed to any given dose (N)/CFU in the control plate (N_0_).

#### Validation of Propidium Monoazide Assay to Detect Viable Microbial Cells

DNA of *Aureobasidium pullulans* MO-28v1 was used to confirm that PMA was intercalating into the fungal DNA and preventing qPCR amplification. Heat sterilization was used to create fungal suspensions containing dead cells. Fungal suspensions (10^6^ cells or conidia per mL) were either subjected or not to heat sterilization for 15 min at 121°C. Subsequently, both sets of samples were treated with PMA (see below) prior to DNA extraction and then extracted DNA from viable/intact cells was quantified with ITS-region qPCR (Checinska Sielaff et al., 2019). Simultaneously, the effect of heat treatment and fungal viability were assessed by cultivation of heat-treated samples and subsequent CFU counts when compared to controls. As expected, PMA treatment resulted in no amplification of the ITS region following heat-sterilization and consequent differentiation between living and dead cells. Negative controls, such as sterile buffers and water were included in all experiments to avoid false-positive results.

#### Initial Sample Processing and DNA Extraction

Both antibiotic-treated and -untreated samples were divided into equal aliquots. One half of each set was treated with PMA dye (final concentration 25 μM) (Biotum, Inc., Hayward, CA) and the other half was left untreated following exactly the steps described elsewhere (Blachowicz et al., 2017). In brief, PMA-treated and untreated samples were kept in darkness for 5 min, followed by 15 min exposure to light in PHaST Blue-Photo activation system for tubes (GenIUL, S.L, Terrassa, Spain). Following dye intercalation, each sample was split in half. One aliquot was subjected to bead-beating for 60 s at 5 m/s on the Fastprep-24 bead-beating instrument (MP Biomedicals, Santa Ana, CA). Subsequently, both bead-beaten and not bead-beaten aliquots were combined and used for DNA extraction using Maxwell 16 Tissue LEV Total RNA purification kit following manufacturer’s instructions (Promega, Madison, WI). As previously reported, the DNA purification was based on magnetic beads capturing and washing off non-DNA materials (Venkateswaran et al., 2012). Extracted and purified DNA was divided into aliquots and stored at −80°C until further analysis.

#### Fungal Community Analysis Using Amplicon Sequencing

Target amplification and sample index PCR reaction: Amplicons were prepared by adding 2 μL of DNA (less than 1 ng/μL) to a PCR mix containing 4X UCP Multiplex Master Mix and 1 μL of each panel pool primer. To cover the six 16S rRNA gene regions and ITS region, three-panel pool primers were used as instructed in the QIAseq16S-ITS kit (QIAGEN, Frederick, MD). The cycling conditions were set to an initial activation step of 95°C for 2 min, followed by 20 cycles of denaturation (95°C, 30 s), annealing (50°C, 30 s), and extension (72°C, 2 min), with a final extension step at 72°C for 7 min. After amplification, the three PCR reactions were combined into a single LoBind tube. PCR products were purified using the QIAGEN QIAseq beads at RT to remove all free unused primers.

Barcoded libraries were generated using QIAseq 16S/ITS Screening Panel kit, which utilizes phased primer technology. To index amplicons using sample indices and Illumina adaptors, 32.5 μL of the amplicons were added to a mix containing 12.5 μL of UCP master Mix and 4 μL of indices. The cycling conditions were set to an initial activation step at 95°C for 2 min followed by 14 cycles of denaturation (95°C, 30 s), annealing (60°C, 30 s), and extension (72°C, 2 min) with a final extension step at 72°C for 7 min. The libraries were purified using two rounds of QIAseq bead cleanup to remove excess adaptors.

##### Library QC and Quantification

The quality of barcoded libraries was determined by electrophoresis using TapeStation Bioanalyzer (Agilent, Santa Clara, CA). Libraries were quantified using QIAGEN QuantArray kit and adjusted to 2 nM, then pooled in equal volume. The pooled amplicons were diluted to 12 pM and sequenced using Illumina MiSeq and the protocol for paired-end (2 × 276 bp) sequencing V3 kit (Illumina, San Diego, CA).

Since the main focus of this study was to characterize the mycobiome, amplicon 16S rRNA sequences were not discussed. ITS sequences obtained from each PMA treated sample were compared with the UNITE database (version 8.1, released 2019-02-02) (Nilsson et al., 2019b) using NCBI BLASTn (version 2.6.0+, parameters: max_target_seqs 5, word_size 5, *e*-value 1e-5) (Altschul et al., 1990). The top hit to the database was retained for each sequence and a table containing the abundance of each ITS amplicon sequence types (STs) in each sample was generated. Canonical correspondence analysis (CCA) (Braak, 1986; Legendre and Legendre, 2020) from the Vegan R package (Oksanen et al., 2013) was used to analyze the distribution of samples with respect to their ST constituents. The variation in distribution among STs was analyzed using the sampling event as a linear predictor. The effect of variation present in control samples was removed prior to analysis.

#### Fungal Diversity Assessed With Shotgun Metagenomics

Samples for metagenome shotgun sequencing were prepared following the steps described previously (Singh et al., 2018). In brief, DNA libraries for shotgun metagenome sequencing were prepared and adapters were added using Nextera Flex DNA Library Preparation Kit from Illumina. The Agilent Fragment Analyzer (Agilent, Santa Clara, CA) was used to check the quality of prepared libraries. Libraries were normalized to 2 nM, pooled, denatured and diluted to 1.8 pM following recommendations by Illumina. The HiSeq 4000 platform (Illumina) was used for sequencing, resulting in 100-bp paired-end reads.

Received reads were trimmed with Trimmomatic (version 0.32) (Bolger et al., 2014) to remove adapters and low-quality ends. Reads shorter than 80 bp were discarded. Remaining reads were normalized (Nayfach and Pollard, 2016) and binned (Singh et al., 2018) to taxonomic levels (domains through species) using the lowest common ancestor (LCA) algorithm by MEGAN 6 (Huson et al., 2016). The samples were analyzed for temporal distribution (sampling 1 and 2) and spatial distribution (locations 1–10 in each cleanroom and differences between geographical locations) to assess succession and persistence using the following statistical approach. Microbial diversity analyses were carried out on normalized reads (∼ 3.1 × 10^8^) and analyses were set to keep at least one unique read to minimize the loss of diversity in low depth samples or for unique reads. BLAST hits of ≥ 20 amino acids and ≥ 90% similarity were collected and used for taxonomic and functional assignments. MEGAN 6 (69) was used for downstream processing and data visualization. The NCBI taxonomy base (Sayers et al., 2009), NCBI-NR protein database were used to assign taxonomic features using DIAMOND (Buchfink et al., 2015) and the weighted LCA algorithm of MEGAN 6 (Huson et al., 2016).

Analysis of similarities (ANOSIM) was carried out using the ANOSIM function from the R package vegan. Mann-Whitney-Wilcoxon (MWW) analyses were performed using the R function wilcox.test and a custom script. Venn diagrams were produced using the R package venneuler and a custom script.

Open access platform Cytoscape (Shannon et al., 2003) was used to perform network analysis and visualize the biogeographical interactions of viable metagenomic reads, while EggNog database (Powell et al., 2012; Huerta-Cepas et al., 2018) was utilized to perform functional analysis of metagenomic reads.

FUNGuild version 1.2 (Nguyen et al., 2016) was used to identify ecological guilds for the most abundant fungal taxa identified in the study. The abundance of members of each guild were summed and used to generate pie charts using GraphPad Prism 9.

#### Controls

To rule out the contamination, all handling and device controls were processed as the collected environmental samples. These controls include sampling devices, sterile PBS used to resuspend collected materials, reagent controls and nucleic acids extraction reagents. Since four different sampling events were carried out, controls were included during each sampling event. Additionally, all samples were processed in the biohood with laminar flow following aseptic techniques. Care was taken to wear proper personal protective equipments and change gloves frequently while working with the samples.

### Data Availability

Amplicon and metagenome data are available under BioProject PRJNA641079, while ITS reads of cultivable isolates are available in GeneBank under MT704867–MT704931 accession numbers.

## Results

### Cultivable Fungal Burden and Diversity

Cultivable fungal populations associated with various locations in two NASA cleanrooms were assessed during two sampling events of each SAF (Supplementary Tables 1, 2). The first sampling at JPL showed fungal counts ranging from 10^2^ to 10^3^ CFU/m^2^; however, cultivable fungi were retrieved from only four of 10 sampled locations. During the second sampling, cultivable fungal counts ranged from 10^1^ to 10^2^ to CFU/m^2^, but fungal populations were observed in all sampled locations (Supplementary Table 1). The ubiquitous isolation of fungi in JPL second sampling might be associated with different activities performed in the JPL-SAF cleanroom between performed samplings. At the time of the first sampling (April 17, 2018), the JPL-SAF was being prepared for Mars 2020 mission (M2020) with minimal human traffic, while more human activities were documented during the second JPL-SAF sampling (September 25, 2018) with rigorous cleaning regime. Fungal burden observed at KSC-PHSF was at levels ∼10^2^ CFU/m^2^ and cultivable populations were retrieved from six and five out of 10 sampled locations (Supplementary Table 2).

Interestingly, using Clipper Mop (CM) to sample ten times more surface area (10 m^2^) yielded similar fungal burden per meter square like for areas sampled with BiSKits or wipes (1 m^2^). Additionally, when extracted samples were enriched with chloramphenicol (100 μg/mL for 24 h.) in the majority of the samples cultivable fungal counts were at the same level as in untreated ones. Only, two locations one from JPL (CM01—ClipperMop sample from location 1) and one from KSC (CM03—ClipperMop sample from location 3) SAF exhibited a onefold increase in fungal burden after antibiotic enrichment (Supplementary Tables 1, 2).

A total of 71 isolates were collected from both SAF and 65 were identified via sequencing the ITS region using NCBI and UNITE databases (Abarenkov et al., 2010), whereas ITS regions from the remaining six isolates were not successfully amplified. In total, 28 distinct species were observed, 16 in the JPL-SAF and 16 in the KSC-PHSF, while 13 isolates were potentially novel species due to low ITS sequence similarity to any known fungal species (Supplementary Table 3). These potentially novel species are subjects of follow up analyses to ascribe their phylogeny. Noteworthy, species identification, based on the ITS region, yielded some discrepancies in the identified species among both databases. Therefore, only when the whole genome is sequenced, annotated, and phenotype is determined, it is possible to definitely ascribe a species. As expected, given the geographic locations and differences in SAF maintenance, only four isolated species *Aureobasidium melanogenum*, *Penicillium fuscoglaucum*, *Penicillium decumbens*, and *Zalaria obscura* were present in both cleanroom facilities. The most prevalent species in the JPL-SAF were *Cladosporium austrohemisphaericum* (5 isolates) and *Arthrocladium tropicale* (3 isolates), while *Aspergillus pseudodeflectus* (7 isolates) and *Talaromyces veerkampii* (3 isolates) were predominant in KSC-PHSF. Forty-two isolates were collected from the samples not treated with antibiotic, while 23 were isolated after antibiotic treatment. Furthermore, when tested for UV-C resistance, 23 of the 65 isolates survived exposure to UV-C dose of 1,000 J/m^2^ (Supplementary Table 3) and showed that UV-C resistance was strain specific. Lastly, neither field nor sampling controls, which underwent the same experimental and processing steps as investigated samples, showed any fungal growth.

### Fungal Diversity Estimated by the Internal Transcribed Spacers Amplicon Sequencing

**FIGURE 1:**
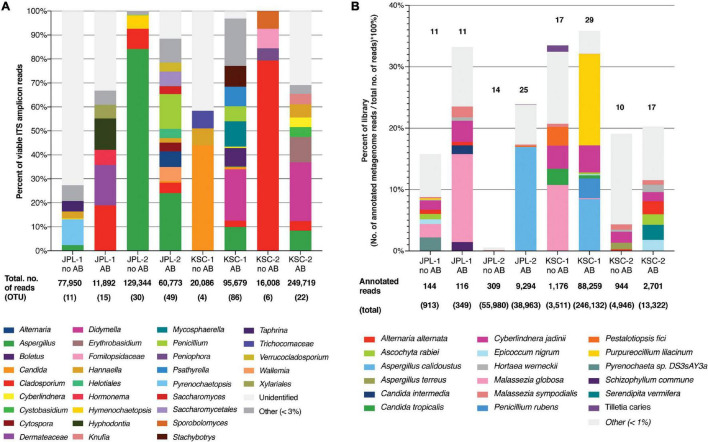
Viable fungal community profiles before and after antibiotic treatment. **(A)** Fungal genera assessed by amplicon sequencing. Numbers of total ITS reads and OTUs for each cleanroom and sampling event are indicated below each bar. **(B)** Fungal species assessed by shotgun metagenomic sequencing. Numbers of total and annotated metagenome reads are listed below each bar. JPL-1 stands for sampling at JPL-SAF on 17 April, 2018; JPL-2 corresponds to the second sampling of JPL-SAF on 25 September, 2018; KSC-1 refers to KSC-PHSF sampling on 12 June, 2018 and KSC-2 corresponds to KSC-PHSF sampling on 24 July, 2018; AB/noAB indicates presence or absence of the antibiotic treatment.

**FIGURE 2:**
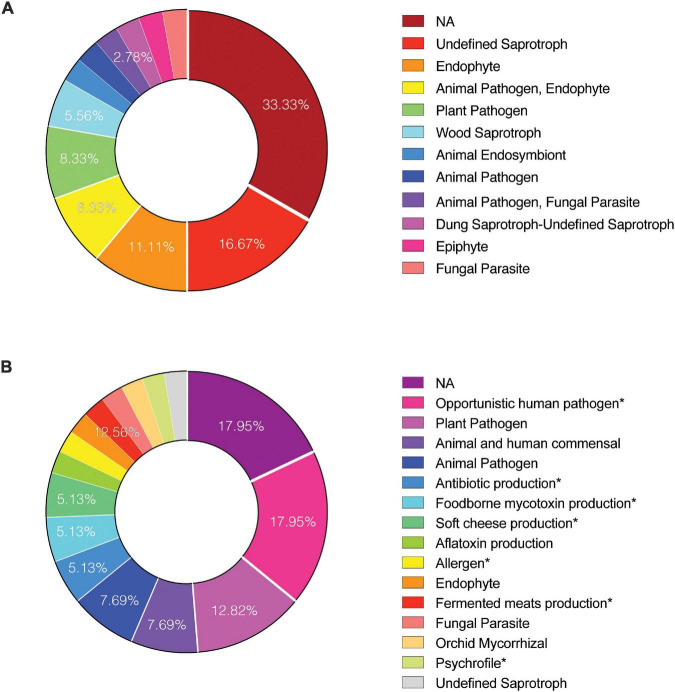
Functional guilds of identified viable fungi. To determine the “ecology” of reported fungal genera and species FUNGuild analysis was performed on **(A)** amplicon and **(B)** metagenome reads, respectively. Identified guild categories are presented. NA, not applicable.

Samples collected from both cleanrooms were assessed for viable/intact (PMA treated) fungal populations in antibiotic-treated and untreated samples (Supplementary Data Set 1). The observed ITS amplicon reads and STs are shown in Figure 1A and Supplementary Figures 3A,B. Overall, higher diversity of viable fungal STs was observed in antibiotic treated samples in both sampled cleanrooms. The number of viable STs in KSC-PHSF was 4 and 6 in untreated samples and 86 and 22 in treated samples during the consecutive samplings. In JPL-SAF STs counts were 11 and 30 in untreated samples and 15 and 49 in treated samples during respective samplings (Supplementary Figure 3A). As expected, the total number of viable reads identified via ITS amplicon sequencing differed among sampled cleanrooms and consecutive sampling events (Supplementary Figure 3B). Noteworthy, the antibiotic treatment showed an impact on observed fungal diversity. The ITS region amplicon sequencing revealed the presence of 32 different fungal genera (Figure 1A and Supplementary Table 4) belonging mostly to phyla Ascomycota (65%) and Basidiomycota (31%). Enrichment with chloramphenicol resulted in retrieval of 5 and 8 STs not observed prior to the treatment at JPL and 11 and 7 STs at KSC during consecutive samplings. Conversely, several STs found prior to antibiotic treatment were not present following chloramphenicol-enrichment (Figure 1A and Supplementary Table 4). Importantly, the STs (with at least 100 reads per ST) extracted from handling and sampling device controls were compared to STs (with at least 100 reads per ST) from surface samples. This analysis showed that the “kitome,” fungal STs innate to sampling devices, did not interfere with the mycobiome analysis of the samples. The STs observed in controls were different kind than the STs extracted from the environment, which was confirmed by GenBank accession numbers (Supplementary Data Set 2). Lastly, to assess the ecology of the identified genera FUNGuild analysis was performed (Figure 2A) revealing that ∼33% of the identified genera were not successfully ascribed to any guild category. However, among the successfully ascribed genera saprotrophs were the most dominant (∼17%) followed by endophytes (∼11), animal pathogens (∼8%), and plant pathogens (∼8).

**FIGURE 3:**
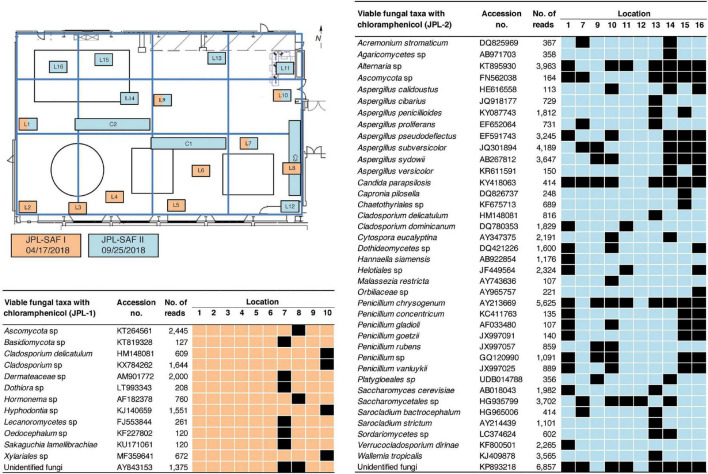
Spatial distribution of viable fungi in samples treated with chloramphenicol at JPL-SAF. JPL-SAF sampling on 17 April, 2018 is marked in orange and on 25 September, 2018 in blue. The locations, marked L1-L16 and C1-C3, indicate areas sampled during the first and second sampling. Locations sampled in the second sampling had to be adjusted to account for on-going M2020 activities. Fungal taxa, GenBank accession #, sampling location, and ITS reads are indicated in the tables. L indicates locations sampled with BiSKit or wipe, while C indicates locations sampled with ClipperMop.

**FIGURE 4:**
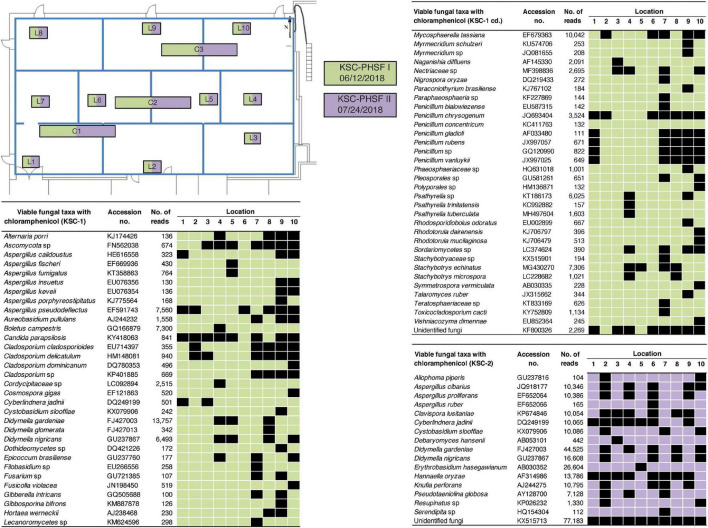
Spatial distribution of viable fungi in samples treated with chloramphenicol at KSC-PHSF. KSC-PHSF sampling on 12 June, 2018 is marked in green and on 24 July, 2018 in violet. A schematic diagram depicts from where samples were collected during both sampling events. Locations are marked as L1–L10 and C1–C3. Fungal taxa, GenBank accession #, sampling location, and ITS reads are indicated in the tables. L indicates locations sampled with BiSKit, while C indicates locations sampled with ClipperMop.

The exact location-wise incidence of observed viable fungal taxa (PMA-treated) in antibiotic enriched samples is presented in Figure 3 for JPL-SAF and Figure 4 for KSC-PHSF. At JPL-SAF viable fungi were observed in only three locations (L7, L8, and L10) during the first sampling (April 17, 2018). However, all tested locations revealed fungal populations during the second sampling (September 25, 2018) as high mission activities were documented. Interestingly, both samplings at KSC-PHSF revealed high abundance of fungal populations at each sampled location (Figure 4).

**FIGURE 5:**
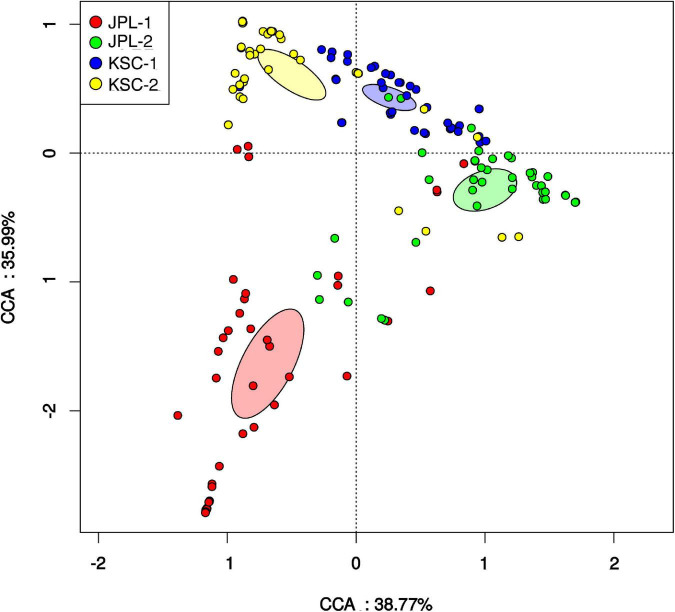
Canonical correspondence analysis (CCA) revealing relationships among fungal populations and associated cleanroom environments. Presented is the distribution of viable fungal taxa in both cleanrooms during consecutive sampling events assessed by amplicon sequencing. Glyphs are colored based on the sampling event. Colored ellipses are included for each sampling event to represent the standard error of the mean of each cluster (99% confidence). CCA axes include the percent variation described by the axis. JPL-1 stands for sampling at JPL-SAF on 17 April, 2018; JPL-2 corresponds to the second sampling of JPL-SAF on 25 September, 2018; KSC-1 refers to KSC-PHSF sampling on 12 June, 2018 and KSC-2 corresponds to KSC-PHSF sampling on 24 July, 2018.

Surface samples were also analyzed based on their ST constituents using CCA (Figure 5), which ordinates samples based on their ST composition similarity. Samples that measured alike ST in similar proportions cluster together, while samples that measured different communities occurred separately in the ordination. The analysis revealed that samples collected during one sampling event at either JPL-SAF or KSC-PHSF cleanrooms tend to cluster together in the CCA ordination and clustered separately from samples collected during different sampling events. Samples collected from KSC (blue and yellow glyphs) cluster independently, but more closely to each other than to the two groups of samples collected from JPL (red and green). Samples collected from the two sampling events at JPL are more disjointed from each other than samples collected from KSC, perhaps representing different community succession patterns in these two cleanrooms.

Lastly, the viable fungal diversity was compared and assessed using ANOSIM and MWW statistics (Table 2). When viable fungal populations were grouped based on the antibiotic treatment, there was a significant difference observed with MWW analysis (*p* = 0.0386), but not ANOSIM (Table 2A). The analysis of viable and antibiotic treated mycobiome constituents of each cleanroom and sampling event revealed some significant differences between JPL-1 vs. JPL-2 (*p* = 0.007) or JPl-1 vs. KSC-1 (*p* = 0.004); however, these may not be definitely ascribed to succession patterns, but rather to an exploratory nature of the study (Table 1B).

**TABLE 2:** ANOSIM and Mann-Whitney-Wilcoxon analyses of viable amplicon reads at genus level of all antibiotic treated and not treated samples **(A)** and samples separated by location and sampling event with chloramphenicol **(B)**.

(A) Antibiotic vs. no antibiotic.					

	MWW	ANOSIM			
*W* = 830.5		*R*: 0.007428			
*p* = 3.86E-02		*p* = 0.274			

**(B) Location and sampling event in samples with chloramphenicol.**					

**Location**	**JPL-1**	**JPL-2**	**KSC-1**	**KSC-2**	
	
JPL-1		*W* = 663	*W* = 720	*W* = 692	
		*p* = 7.35E-03	*p* = 2.79E-02	*p* = 1.57E-02	
	
JPL-2	*R* = 0.2594		*W* = 1038	*W* = 1023.5	**MWW**
	*p* = 0.007		*p* = 5.48E-01	*p* = 6.37E-01	
	
KSC-1	*R* = 0.2668	*R* = 0.012111		*W* = 1043	
	*p* = 0.004	*p* = 0.316		*p* = 5.21E-01	
	
KSC-2	*R* = 0.04844	*R* = 0.02089	*R* = 0.07022		
	*p* = 0.2	*p* = 0.323	*p* = 0.104		
	
		**ANOSIM**			

**FIGURE 6:**
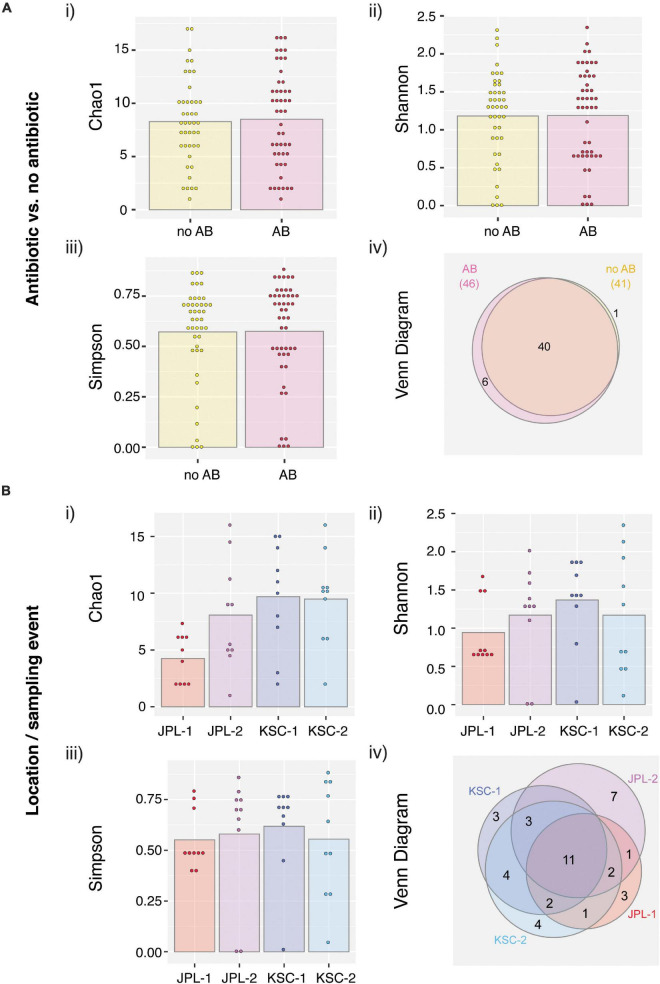
Viable fungal taxa ordinate analysis of samples from JPL-SAF and KSC-PHSF. **(A)** To determine the impact of the chloramphenicol treatment on the fungal diversity, normalized amplicon reads of antibiotic treated and untreated samples were analyzed. Tested indices include **(i)** Chao1 diversity analysis **(ii)** Shannon index **(iii)** Simpson index, and **(iv)** Venn diagram. **(B)** Location/sampling event-based diversity was assessed using amplicon reads of antibiotic treated samples only. Tested indices include **(i)** Chao1 diversity analysis, **(ii)** Shannon index, **(iii)** Simpson index, and **(iv)** Venn diagram showing the distribution of viable fungal taxa. JPL-1 stands for sampling at JPL-SAF on 17 April, 2018; JPL-2 corresponds to the second sampling of JPL-SAF on 25 September, 2018; KSC-1 refers to KSC-PHSF sampling on 12 June, 2018 and KSC-2 corresponds to KSC-PHSF sampling on 24 July, 2018; AB/noAB indicates presence or absence of the antibiotic treatment.

All PMA treated viable/intact samples, both with and without chloramphenicol treatment, were assessed for alpha and beta diversity using various microbial diversity indices, including Chao1 (Figure 6Ai), Shannon (Figure 6Aii), Simpson (Figure 6Aiii), and Venn diagrams showing species overlap (Figure 6Aiv). The abundance-based Chao1 estimator of alpha diversity showed slight increase in the fungal diversity in chloramphenicol treated samples. The Shannon estimator, which measures species richness and the Simpson estimator, which measures species evenness, revealed no differences upon chloramphenicol treatment. Lastly, the Venn diagram, which reveals the presence of unique species within each location and sampling event showed that only six different species were found in chloramphenicol-treated samples when compared to the chloramphenicol-untreated samples. In addition, the alpha and beta diversity were assessed location-wise for samples treated with antibiotic (Figure 6B). The observed differences in the indices confirmed no clear pattern in fungal succession within each sampled SAF (Figure 6B). Venn Diagram showed variations among sampled locations, which most likely stem from the exploratory nature of the study and geographic separation of the cleanrooms.

### Fungal Diversity Estimated by Metagenome Shotgun Sequencing

After normalization across all samples, chloramphenicol- and PMA-treated or untreated (*n* = 192), the metagenomic analysis yielded ∼4.8 × 10^8^ reads associated with microorganisms. Out of these reads, only the reads from viable and intact cells (reads from PMA treated samples) were the focus of this study (Supplementary Data Set 3). At the domain level 92.52% of the reads were bacterial, 7.43% eukaryotic, while both viral (0.02%) and archaeal (0.04%) reads were negligible. Such a distribution of metagenomic reads remains in sharp contrast to other systems, including the ISS, homes and offices where about 30–50% of the reads belong to eukaryotes (Singh et al., 2018). Among the 3.6 × 10^7^ eukaryotic reads detected, only 2.1 × 10^6^ belonged to fungi and were further characterized and are presented in this manuscript. Furthermore, about 13–35% of all reads obtained from both cleanrooms were successfully annotated, with the exception of samples collected from JPL during the second sampling and not treated with chloramphenicol, where only 1% of the reads were annotated (Figure 1B and Supplementary Figure 3C).

**FIGURE 7:**
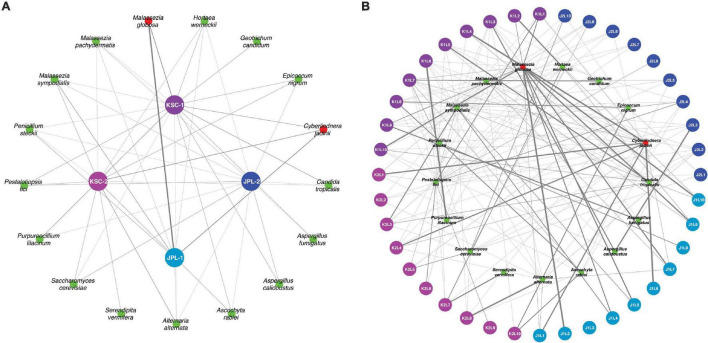
Network analysis presenting biogeographical distribution of viable fungal populations after antibiotic treatment. **(A)** Presented are the most abundant (abundance > 0.01% of all reads) fungal species in JPL-SAF and KSC-PHSF. **(B)** The most abundant fungal reads are assigned to sampled locations. The thickness and transparency of the edges (lines) follows the calculated e-weights. JPL-1 stands for sampling at JPL-SAF on 17 April, 2018; JPL-2 corresponds to the second sampling of JPL-SAF on 25 September, 2018; KSC-1 refers to KSC-PHSF sampling on 12 June, 2018 and KSC-2 corresponds to KSC-PHSF sampling on 24 July, 2018. In **(B)**, sampling locations are marked using the system that indicates sampled cleanroom at JPL or KSC marked as J or K, first or second sampling event indicated by 1 or 2, followed by sampled location labeled as L1–L10, for example, J1L2 stands for JPL-SAF first sampling at location 2. The red colored taxa were more abundant in control samples.

Metagenomic fungal reads were dominated by Ascomycota (91%) and Basidiomycota (7.15%); however, representative reads of Blastocladiomycota, Chytridiomycota, Cryptomycota, Mucoromycota, and Zoopagomycota were also retrieved. Similar to amplicon sequencing (Figure 1A and Supplementary Figure 3B), the total number of fungal reads differed among analyzed samples. In total, 32 fungal species were identified using shotgun metagenome sequencing. The number of viable fungal species differed among antibiotic untreated and treated samples. At KSC-PHSF it was, respectively, 10 and 17 identified species during the first sampling and 10 and 17 during the second sampling. At the JPL-SAF, the difference was observed during the second sampling only, when the number of species changed from 14 to 25, while the number of species remained the same during the first sampling (Figure 1B and Supplementary Table 5). Among the most dominant species at both cleanrooms were *Malassezia globosa* (human skin and hair-associated fungus) and *Cyberlindnera jadinii* (teleomorph of *Candida utlilis*). Noteworthy, the antibiotic treatment resulted in retrieving *Aspergillus calidoustus* and other *Aspergillus* species at JPL-SAF, and *Penicillium* species, *Purpureocillium lilacinum*, *Alternaria alternata, Epicoccum nigrum, and Serendipita vermifera* at KSC-PHSF when compared to untreated samples (Supplementary Table 5). To visualize the biogeography of isolated strains the network analysis was computed for chloramphenicol-treated samples (Figure 7A). This analysis confirmed higher abundances of *M. globosa* and *C. jadinii* marked with thicker edges as oppose to less abundant isolates represented with finer edges. Detected reads were also assigned to specific sampling locations within the JPL-SAF and KSC-PHSF (Figure 7B). Overall metagenomic reads retrieved from handling and sampling device controls were scarce. However, in case of observing more abundant reads in controls, the matching ST reads in the samples were excluded from downstream analyses (*M. globosa* JPL-2 and KSC-2 and *C. jadinii* JPL-2) (Supplementary Data Set 4). The insight into “ecology” of reported species assessed either via FUNGuild or literature search (Figure 2B) revealed that ∼18% of identified species were not successfully ascribed to any guild category. Among the ascribed categories opportunistic pathogens (∼18), plant pathogens (∼13%), animal and human commensals (∼8%), and animal pathogens (∼8%) were predominant.

Similar to ITS amplicon reads, viable fungal diversity at the species level estimated by the shotgun metagenomic was analyzed using ANOSIM and MWW statistics (Table 3). When viable fungal populations were grouped based solely on the antibiotic treatment, there was a significant difference observed with ANOSIM analysis, but not in MWW (Table 2A). When PMA and antibiotic treated mycobiome constituents of each cleanroom and sampling event were pooled, some statistical differences were observed amongst the locations, but again these not revealed any succession patterns just the expected distinction related to geographical separation (Table 2B).

**TABLE 3:** ANOSIM and Mann-Whitney-Wilcoxon analyses of viable metagenome reads at species level of all antibiotic treated and not treated samples **(A)** and samples separated by location and sampling event with chloramphenicol **(B)**.

(A) Antibiotic vs. no antibiotic.					

MWW		ANOSIM
*W* = 124		*R*: −0.01645			
*p* = 1.989E-10		*p* = 0.881			

**(B) Location and sampling event in samples with chloramphenicol.**					

**Location**	**JPL-1**	**JPL-2**	**KSC-1**	**KSC-2**	

JPL-1		*W* = 229	*W* = 36.5	*W* = 466	
		*p* = 2.57E-08	*p* = 9.495E-14	*p* = 1.16E-03	
	
JPL-2	*R* = 0.2734		*W* = 323	*W* = 995.5	**MWW**
	*p* = 0.004		*p* = 1.245E-05	*p* = 1.77E-02	
	
KSC-1	*R* = 0.08628	*R* = −0.08178		*W* = 1302	
	*p* = 0.061	*p* = 0.842		*p* = 5.56E-08	
	
KSC-2	*R* = 0.1259	*R* = 0.1002	*R* = 0.1557		
	*p* = 0.032	*p* = 0.124	*p* = 0.052		
	
		**ANOSIM**			

**FIGURE 8:**
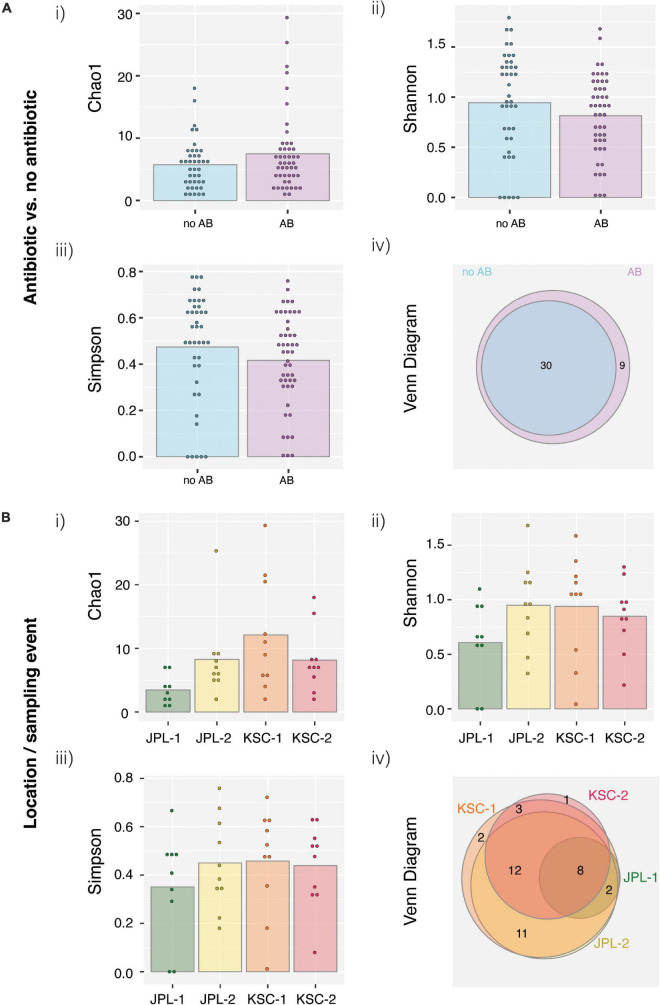
Species-level ordinate analysis of samples from JPL-SAF and KSC-PHSF cleanrooms. **(A)** To determine the impact of the chloramphenicol treatment on the fungal diversity, normalized metagenomic reads of antibiotic treated and untreated samples were analyzed. Tested indices include **(i)** Chao1 diversity analysis **(ii)** Shannon index **(iii)** Simpson index, and **(iv)** Venn diagram. **(B)** Location/sampling event-based diversity was assessed using metagenomic reads of antibiotic treated samples only. Tested indices include **(i)** Chao1 diversity analysis, **(ii)** Shannon index, **(iii)** Simpson index, **(iv)** Venn diagram showing the distribution of viable fungal taxa. JPL-1 stands for sampling at JPL-SAF on 17 April, 2018; JPL-2 corresponds to the second sampling of JPL-SAF on 25 September, 2018; KSC-1 refers to KSC-PHSF sampling on 12 June, 2018 and KSC-2 corresponds to KSC-PHSF sampling on 24 July, 2018; AB/noAB indicates presence or absence of the antibiotic treatment.

All viable samples (PMA treated), with and without chloramphenicol treatment, were assessed for alpha and beta diversity (Figure 8A). The Chao1 estimator showed an increase in the fungal diversity in chloramphenicol treated samples (Figure 8Ai). In contrast, the Shannon and Simpson indicators revealed a decrease in fungal diversity upon chloramphenicol treatment (Figures 8Aii,iii). Lastly, Venn diagrams showed that only nine species were unique for chloramphenicol-treated samples when compared to the untreated ones (Figure 8Aiv). The fungal diversity assessed for locations using antibiotic-treated samples showed no definite patterns allowing for assessing fungal diversity in consecutive samplings, as the observed trends were opposite in sampled cleanrooms (Figure 8B). Lastly, Venn diagrams showed that eight species—namely *Alternaria alternata, Aspergillus sydowii, Cyberlindnera jadinii, Hortaea werneckii, Malassezia globosa, M. pachydermatis, M. sympodialis*, and *Penicillium steckii*—were common to all sampling events and the locations in antibiotic treated samples (Figure 8Biv).

**FIGURE 9:**
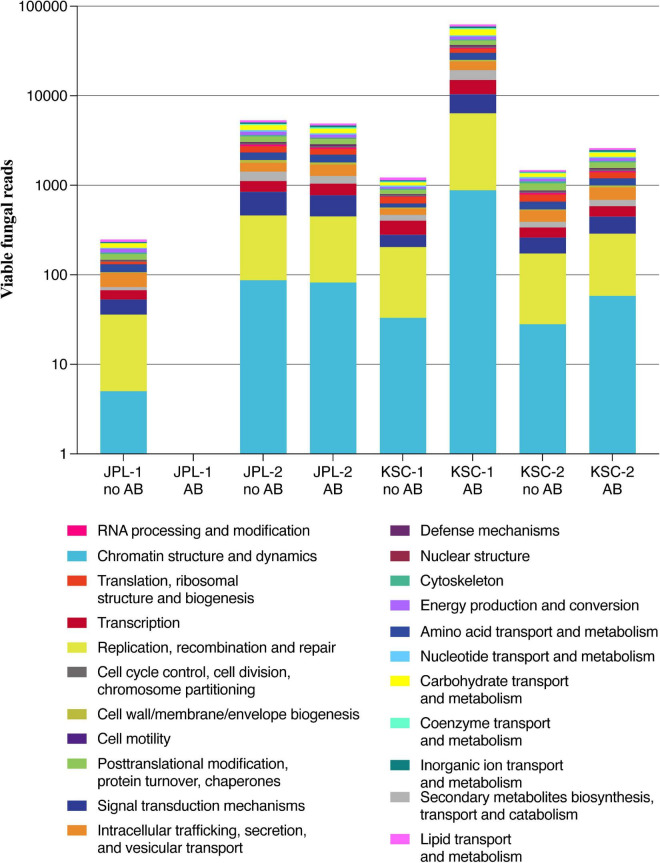
Analysis of metagenomic reads from JPL-SAF and KSC-PHSF cleanrooms. Presented are the most abundant genes/proteins in both cleanrooms during consecutive sampling events assessed by shotgun metagenomics. JPL-1 stands for sampling at JPL-SAF on 17 April, 2018; JPL-2 corresponds to the second sampling of JPL-SAF on 25 September, 2018; KSC-1 refers to KSC-PHSF sampling on 12 June, 2018 and KSC-2 corresponds to KSC-PHSF sampling on 24 July, 2018; AB/noAB indicates presence or absence of the antibiotic treatment.

Noteworthy, characterization of metagenomic reads revealed the prevalence of genes involved in processes related to chromatin structure and dynamics, replication, recombination and repair and signal transduction mechanisms in all samples except JPL-1 treated with chloramphenicol (Figure 9 and Table 4). When chloramphenicol-treated samples were compared to untreated ones the change in transporter proteins (ENOG410XNQK, COG1131, COG1132) was observed (Table 4). However, metatranscriptomic analysis is required to confirm dynamic changes in gene expression in response to the treatment with antibiotic.

**TABLE 4:** Functional analysis of viable metagenome reads of all antibiotic treated and not treated samples.

EggNog	Putative function/activity	JPL-1 no AB	JPL-1 AB	JPL-2 no AB	JPL-2 AB	KSC-1 no AB	KSC-1 AB	KSC-2 no AB	KSC-2 AB
ENOG410XNQK	Transporter	4	0	77	87	5	1,647	32	31
COG0477	Major facilitator Superfamily	6	0	65	57	10	1,369	11	29
COG2801	Retrotransposon protein	0	0	5	8	52	1,377	5	4
COG2124	Cytochrome p450	0	0	99	68	12	994	13	30
COG3321	Synthase	0	0	27	29	13	944	4	15
COG1020	Non-ribosomal peptide synthetase	0	0	31	20	4	774	2	4
COG0531	Amino acid	6	0	37	23	0	561	15	13
COG1131	(ABC) transporter	0	0	37	37	9	532	0	17
COG1132	(ABC) transporter	0	0	39	50	6	445	11	20
COG0474	P-type ATPase	2	0	20	17	12	399	7	18
COG5032	Phosphatidylinositol kinase	3	0	25	30	9	376	8	22
COG1472	Hydrolase family 3	0	0	35	17	14	334	4	2
COG1012	Dehydrogenase	0	0	11	20	1	355	2	15
COG2303	Oxidoreductase	7	0	16	17	0	333	8	17
COG0654	Xidoreductase	2	0	19	19	6	317	2	10
COG2072	Monooxygenase	0	0	9	10	6	338	1	2
COG0366	Alpha amylase, catalytic	2	0	17	12	0	306	5	9
COG0464	AAA ATPase	1	0	25	40	0	269	8	5
COG0604	Alcohol dehydrogenase	0	0	18	11	0	284	2	10
COG1215	Glycosyl transferase, family	0	0	42	30	6	211	1	14
COG1112	Helicase	3	0	14	30	0	247	1	8
COG0277	FAD linked oxidase	0	0	6	9	4	258	2	11
COG1643	Helicase	1	0	43	36	4	189	4	10
COG0318	Amp-dependent synthetase and ligase	2	0	31	22	0	214	5	7
COG5059	Kinesin family member	0	0	8	12	9	235	7	6
ENOG410XRBH	SRSF protein kinase	0	0	27	11	0	235	0	3
COG3320	Domain protein	1	0	16	4	8	230	3	0
COG1501	Hydrolase, family 31	0	0	6	8	1	238	2	6
COG2272	Carboxylesterase	0	0	6	5	0	243	0	1
ENOG410XNPJ	Polyketide synthase	0	0	1	2	7	240	0	2

## Discussion

Successful isolation of fungi is necessary for determining and characterizing their unique molecular features and biotechnological value, which might enable development of countermeasures (Knox et al., 2016; Romsdahl et al., 2018b,^2020^; Urbaniak et al., 2019). Therefore, seeking for more efficient isolation methods of fungi from the cleanroom environments is critical. In this study chloramphenicol was paired-with PMA treatment to assess the amplifying effect on fungal isolation. In the course of the study 13 potentially novel fungal species have been isolated, but only six were unique to the chloramphenicol-treated samples, showing moderate benefit of antibiotic use. More importantly, characterization of these potentially novel species might lead to the understanding of how fungi thrive in harsh, oligotrophic environments. Interestingly, despite using growth media containing varying dose of the chloramphenicol (DRBC vs. PDA) no differences in isolated fungal species nor abundance were observed. Among cultured fungi, *Penicillium, Aspergillus*, and *Cladosporium* species were predominant, which were also reported on the Skylab (Brockett et al., 1978), Mir Space Station (Alekhova et al., 2005), Japanese ISS module “KIBO” (Satoh et al., 2016), and ISS high-efficiency particle air filters (HEPA) debris (Checinska et al., 2015). In contrast, ISS environmental surfaces near where humans dwell on the spacecraft, were dominated by *Rhodotorula* and other members of *Sporiodiobolaceae* (Checinska Sielaff et al., 2019). Fungal burden assessed by ITS amplicon sequencing revealed variations in fungal abundance at JPL-SAF during increased mission activity, suggesting humans as vectors and the source of the contamination. Such correlation between human presence and changes in fungal populations in confined spaces has been previously reported (Checinska et al., 2015; Blachowicz et al., 2017; Checinska Sielaff et al., 2019).

One of the main goals of the study was to overcome the overwhelming abundance of bacterial populations, which overshadow the fungal presence, while performing molecular analyses. Pairing the antibiotic and PMA treatment with targeted amplification revealed presence of rare mycobiome (Dolan, 1971; Nilsson et al., 2019a). However, the positive impact of the antibiotic itself on retrieved fungal diversity was not definitive, which may be related to the fact that chloramphenicol is considered bacteriostatic rather than bactericidal (Oong and Tadi, 2022). Even though no mission-related activities or human traffic were documented at KSC-PHSF, the cleanroom was maintained to the ISO-7 certification requirements. The ISO-7 cleanroom classification must have less than 352,000 particles > 0.5 micron per cubic meter and 60 HEPA filtered air changes per hour. Interestingly, irrespective of human traffic and stringent maintenance, there was high fungal diversity during both samplings. Substantial differences in fungal abundance in both JPL and KSC cleanrooms might be attributed to the exploratory nature of the study and several other factors, like different geographical locations, climates (relative humidity) and in-house cleaning procedures used to maintain the cleanrooms. The analysis of the STs using CCA showed that samples collected during the same sampling event tend to cluster together and separately from samples collected during different sampling events in both cleanrooms. This might be due to the biogeographical attributes (Moissl et al., 2007) corresponding to each facility since human traffic related to mission activities and the exchange of clean air into the facility were occurring. In addition, differences in spatial distribution of constituents of the mycobiome was documented. For instance, JPL-SAF location L1 had no viable fungi reported after the first sampling, while high fungal abundance were observed during the second sampling. This may be related to the fact that the location L1 is the only entry point for cleanroom personnel, while the L12 location, which showed extremely low fungal presence, was not heavily used during on-going M2020 activities. Similarly, increased human traffic was documented in locations L13–L16, which corresponds with high fungal presence. Inversely, other sampled locations (L7–L11) exhibited both lower human traffic and lower fungal abundance. This was of no surprise since fungal communities have been reported to be affected by human presence in a simulated closed habitat (Blachowicz et al., 2017) and aboard the ISS (Checinska Sielaff et al., 2019).

One of the aspects that is crucial to address is how the fungal diversity observed in cleanrooms corresponds to the fungal populations in other confined spaces. So far, the majority of the available reports were based on the cultivable approach, while culture-independent mycobiome analyses are scarce. When the mycobiome of the simulated closed habitat ILMAH was assessed during 30-day occupation by student astronauts, the dominant genera included *Epicoccum, Alternaria*, and family *Pleosporaceae* (Blachowicz et al., 2017), while in the spacecraft assembly cleanrooms the most dominant members were *Aspergillus, Cladosporium*, and *Didymella* (La Duc et al., 2012). Interestingly, the ecological association of fungi reported in JPL-SAF and KSC-PHSF revealed dominance of saprothrophs, endophytes, and animal pathogens. In contrast to fungal populations usually associated with the natural outdoor environment in ILMAH and examined cleanrooms, the surveillance of the ISS surfaces revealed the presence of fungi associated more with humans rather than with outdoor-associated environments (Checinska Sielaff et al., 2019). Similarly, fungi isolated from neonatal environmental surfaces of intensive care units composed of different kinds of fungi than observed in the cleanrooms with overwhelming presence of *Candida* and *Saccharomyces* (Heisel et al., 2019). The facility-specific fungal composition might be attributed to the cleaning management and system engineering of the controlled environment.

Metagenomic analysis of collected samples provided higher resolution for species identification. Eight identified species were present in both cleanrooms revealing that despite the geographical distance certain core species remain the same, including species of environmental origins (*Alternaria alternata*, *Aspergillus sydowii)* and human commensals (*Malassezia globosa, M. pachydermatis, M. sympodialis*). Noteworthy, the mycobiome associated with environmental surfaces in the crewed ISS was dominated by *Penicillium* species and *Aspergillus calidoustus* (Singh et al., 2018), while high abundance of *Cyberlindnera jadinii* was reported on the skin of astronauts (Sugita et al., 2016). Species identification via shotgun metagenomics presents an opportunity to employ these fungi as model organisms to develop technologies aiming at microbial reduction and eradication especially because ecological analysis showed that some of the reported fungi might be opportunistic human pathogens. The dominance of *C. jadinii* on the oligotrophic cleanroom surfaces requires further investigation. This biotechnologically interesting yeast was previously tested for the production of its biomass/protein on lignocellulosic sugars and nitrogen-compounds as an alternative to plant or animal fed (Lapeña et al., 2020). Considering that discovery, *C. jadinii* could potentially nourish growth of other microorganisms found in closed habitats. In addition, understanding the mechanisms of resistance to very high doses of UV-C (Valero et al., 2007) and identifying genes and secondary metabolites involved in the survival of fungi following exposure to simulated Mars conditions and UV-C (Blachowicz et al., 2019a,^2020^) might foster developing appropriate cleaning regimens. Lastly, the analysis of metagenomic reads in this study showed high abundance of transporter and retrotransposon proteins as well as cytochrome P450. These proteins were reported to contribute to improving the fungal fitness and fostering fungal adaptation and survival when exposed to harsh environmental niches (Sandmeyer and Clemens, 2010; Kulikova-Borovikova et al., 2018; Shin et al., 2018). Only by conducting such in depth phenotypic analyses on isolates found in SAF environments can PP investigators tailor the countermeasures to improve prevention of forward contamination.

Despite the incidence of fungi in SAF environments (La Duc et al., 2012; Weinmaier et al., 2015), no NASA studies have assessed the extent to which these microorganisms are resistant to, or grow under, space conditions and might potentially survive and withstand existing PP microbial reduction and cleaning approaches (Rummel et al., 2002). Importantly, recent studies have shown that representatives of *Cladosporium* and *Aspergillus* survive exposure to simulated Mars conditions and space radiation (Blachowicz et al., 2019a; Cortesão et al., 2020). Hence, the results generated in this study might aid in controlling the potential for forward contamination of fungi through robotic and crewed missions. The results of this study also directly respond to the National Research Council (NRC) recommendations for the Prevention of the Forward Contamination of Mars, that suggest examining the survival of microorganisms under adverse space conditions to assess the microbial potential to survive on spacecraft *en route* to other planetary bodies (National Research Council, 2006). Furthermore, fungal persistence on spacecraft associated surfaces pose a health threat to astronauts, as some fungal strains are pathogenic. One example is, *Aspergillus fumigatus*, a species ubiquitous in the environment, but shown to be more virulent when exposed to space conditions on the ISS than known clinical isolates (Knox et al., 2016). More importantly, *A. fumigatus* conidia have survived exposure to simulated Mars conditions for 30 min, underscoring its potential to survive in environmental extremes, including space (Blachowicz et al., 2019a). Thus, fungi isolated in this study that survived exposure to UV-C 1,000 J/m^2^ are of paramount importance for conducting further studies to assess their capability to resist and survive exposure to various space conditions, including X-rays or cosmic radiation. Such understanding would promote both development of appropriate countermeasures and containing fungal forward contamination during long-duration interplanetary exploration.

While endospore-forming bacteria and halophilic archaea are known to survive and even proliferate under extreme desiccation or hypersaline conditions, it is fungi that are capable of thriving and proliferating in low water activity (a_w_) environments. The lowest a_w_ limit where fungal growth was reported is 0.62 (Beuchat and Scouten, 2002); hence, fungal spores and conidia may survive and germinate at somewhat lower water activity (Wheeler and Hocking, 1988). Investigators have also pointed out that manipulating the accumulated intracellular polyol content can extend the range of water availability over which fungal propagules can germinate (Hallsworth and Magan, 1995). Fungi have been traditionally thought of as mostly mesophilic, heterotrophic organisms; however, eukaryotes isolated from polar ice samples are mostly fungal spores and conidia (Vorobyova et al., 2001). More than that, several fungi exhibiting radiotropism (growth toward the radiation source) were isolated from Chernobyl Nuclear Power Plant accident sites (Zhdanova et al., 2004; Blachowicz et al., 2019a). In light of these reports and considering the current interest in astrobiology and PP implications, systematical investigation of fungal presence in NASA associated cleanrooms is of high value.

To summarize, presence of fungi in habitats, even when in low abundance in comparison to bacterial counts, should not be neglected. Fungi introduced to cleanrooms and other built environments with human presence might potentially pose a threat to the closed habitats (biocorrosion) as well as their immunocompromised occupants (pathogenicity), and more critically, to the pristine environments of other celestial bodies during interplanetary explorations. This study was a first step to decipher cultivable, viable and total fungal populations in spacecraft assembly facilities and to evaluate the “real” potential of fungi encountered as biological contaminants. Furthermore, the outcomes of such studies could be translated and implemented in other cleanrooms requiring reduced counts of problematic resilient fungal populations, like intensive care units, operating rooms, or cleanrooms in the semiconducting and pharmaceutical industries.

## Data Availability Statement

Amplicon and metagenome data are available under BioProject PRJNA641079, while ITS reads of 593 cultivable isolates are available in GeneBank under accession numbers MT704867–MT704931.

## Author Contributions

KV designed the study, interpreted the data, and co-drafted the manuscript. AB wrote the manuscript, generated figures, and contributed to data analysis and interpretation. SM collected, processed the samples, and generated all the presented results. NS processed and analyzed the metagenomic data. JW processed, analyzed the amplicon sequencing data, and performed statistical analyses. CP performed cultivable species identification. CL collected the samples, drafted corresponding parts of the manuscript, and provided sample location figure. DB and CM performed shotgun metagenomic sequencing. All authors read and approved the final manuscript.

## Conflict of Interest

The authors declare that the research was conducted in the absence of any commercial or financial relationships that could be construed as a potential conflict of interest.

## Publisher’s Note

All claims expressed in this article are solely those of the authors and do not necessarily represent those of their affiliated organizations, or those of the publisher, the editors and the reviewers. Any product that may be evaluated in this article, or claim that may be made by its manufacturer, is not guaranteed or endorsed by the publisher.
