# Supplementary material for: The Isolation and Characterization of Rare Mycobiome Associated With Spacecraft Assembly Cleanrooms
Source: Front Microbiol. 2022 Apr 26;13:777133. doi: 10.3389/fmicb.2022.777133 (PMC9087587; doi:10.3389/fmicb.2022.777133)
Supplement: Supplementary file 1 [file Table_1.PDF]

Supplementary Table 1. Cultivable fungal burden of JPL-SAF. **A)** Sampling on 04/17/2018 **B)** Sampling on 09/25/2018.

A)

Sample	JPL-1 CFU/m^2	
	PDA	DRBC
L1		
L2		5.00E+02
L3	1.00E+03	
L4		
L5		
L6		
L7	5.00E+02	1.00E+03
L8		
L9	5.00E+02	
L10		
NC		
HC		

B)

Sample	JPL-2 CFU/m^2			
	PDA no AB	PDA AB	DRBC no AB	DRBC AB
L1	1.95E+02	1.50E+01		
L7	1.50E+02	9.00E+01		
L9	3.00E+01	9.00E+01	1.50E+01	6.00E+01
L10	1.50E+02	6.00E+01	9.00E+01	1.05E+02
L11	1.35E+02			
L12	1.50E+01			
L13	3.00E+01	6.00E+01	3.00E+01	3.00E+01
L14	2.10E+02	9.00E+01		
L15	9.00E+01	7.50E+01		1.05E+02
L16	4.50E+01	3.00E+01		
HC				
NC				
CM01	9.00E+01	1.35E+02	1.50E+01	
CM02	4.50E+01	1.50E+01	1.50E+01	
CM03	2.10E+02	2.25E+02	1.35E+02	4.50E+01
CMHC				
CMDC				

PDA – Potato Dextrose Agar
DRBC - Dichloran Rose Bengal Chloramphenicol Agar
L – Location
CM – ClipperMop
HC/DC – Handling control / Sampling device control
