# Supplementary material for: The Isolation and Characterization of Rare Mycobiome Associated With Spacecraft Assembly Cleanrooms
Source: Front Microbiol. 2022 Apr 26;13:777133. doi: 10.3389/fmicb.2022.777133 (PMC9087587; doi:10.3389/fmicb.2022.777133)
Supplement: Supplementary file 2 [file Table_2.PDF]

Supplementary Table 2. Cultivable fungal burden of KSC-PHSF. Sampling KSC-1 was conducted on 06/12/2018, while sampling KSCp-2 was performed on 07/17/2018.

Sample	KSC-PHSF CFU/m^2							
	**KSC-1**		**KSC-2**		**KSC-1**		**KSC-2**	
	PDA no AB	PDA AB	PDA no AB	PDA AB	DRBC no AB	DRBC AB	DRBC no AB	DRBC AB
L1		6.00E+01	6.00E+01	2.40E+02			6.00E+01	6.00E+01
L2								
L3	3.45E+02	2.55E+02	7.50E+02	2.85E+02	6.45E+02	2.55E+02	8.55E+02	3.60E+02
L4	6.00E+01	6.00E+01						
L5								
L6							6.00E+01	6.00E+01
L7	6.00E+01	1.20E+02	6.00E+01		6.00E+01	2.40E+02	6.00E+01	
L8	1.00E+02	9.00E+01	1.20E+02	1.20E+02	1.20E+02	6.00E+01		
L9						6.00E+01		
L10				6.00E+01			6.00E+01	
HC								
NC								
CM01			6.00E+01					
CM02			2.40E+02	1.20E+02	6.00E+01	6.00E+01	1.80E+02	9.00E+01
CM03	6.00E+01		1.05E+02	4.50E+02			6.00E+01	1.20E+02
CMHC								
CMDC								

PDA – Potato Dextrose Agar
DRBC - Dichloran Rose Bengal Chloramphenicol Agar
I/II – Sampling event
L – Location
CM – ClipperMop
HC/DC – Handling control / sampling device control
