# Supplementary material for: The Isolation and Characterization of Rare Mycobiome Associated With Spacecraft Assembly Cleanrooms
Source: Front Microbiol. 2022 Apr 26;13:777133. doi: 10.3389/fmicb.2022.777133 (PMC9087587; doi:10.3389/fmicb.2022.777133)
Supplement: Supplementary file 3 [file Table_3.PDF]

Supplementary Table 3. ITS identification of isolates collected from JPL-SAF and KSC-PHSF cleanrooms

Species	GenBank Accession No.	% Identity	UNITE Identification	UNITE Reference	UNITE % Identity	N Isolates	Strain #				
Cladosporium austrohemisphaericum	NR_152289.1	97%	*Cladosporium*	HQ608074	97%	5	FJI-L7-BK-DG1	FJII-L8-SW-PAB1	FJI-L7-BK-P1	FJII-L5-SW-P2	FJII-L6-SW-P2
Neocatenulostroma abietis	NR_145103.1	99%	*Neocatenulostroma germanicum*	KR995100	99%	1	FJI-L3-BK-P2				
Penicillium sumatraense	NR_119812.1	99%	*Penicillium sumatraense*	MK910053	100%	2	FJI-L3-BK-DG1	FJI-L10-BK-P2			
Roussoella neopustulans	NR_155715.1	94%	*Massarina igniaria*	MW081388	100%	1	FJI-L9-BK-P1				
Oidiodendron eucalypti	NR_160624.1	92%	Helotiales	JX657347	100%	1	FJI-L2-BK-P1				
Trichoderma citrinoviride	NR_077178.1	99%	*Trichoderma citrinoviride*	MW085071	99%	2	FJI-L10-BK-P1	FJI-L10-BK-P3			
Epicoccum keratinophilum	NR_158278.1	100%	*Epicoccum keratinophilum*	LT592928	100%	2	FJII-L3-CM-DR4	FJII-L3-CM-PAB3			
Penicillium fuscoglaucum	NR_163669.1	100%	*Penicillium commune*	MK660354	100%	1	FJII-L5-SW-P3				
Neophaeococcomyces aloes	NR_132069.1	98%	*Neophaeococcomyces aloes*	KF777182	98%	1	FJII-L3-CM-P2				
Zalaria obscura	NR_153466.1	99%	*Zalaria obscura*	KX579096	100%	3	FJII-L3-CM-DR3	FKI-L7-BK-DRAB2	FKI-L7-BK-DAB3		
Aspergillus pseudoglaucus	NR_135336.1	99%	*Aspergillus amstelodami*	KX090347	99%	1	FJII-L3-SW-P1				
Fusarium nygamai	NR_130698.1	100%	*Fusarium annulatum*	MN548436	100%	1	FJII-L4-SW-PAB2				
Penicillium fuscoglaucum	NR_163669.1	98%	*Penicillium fuscoglaucum*	MT558936	98%	3	FKI-L3-CM-P1	FJII-L4-SW-DR1	FJII-L8-SW-P1		
Penicillium kongii	NR_138336.1	99%	*Penicillium brevicompactum*	MH857204	100%	1	FJII-L9-SW-P1				

Lecanicillium kalimantanense	NR_121200.1	93%	*Engyodontium album*	LT549076	100%	1	FJII-L10-SW-P1						
Aureobasidium melanogenum	NR_159598.1	99%	*Aureobasidium melanogenum*	MT781960	100%	1	FJII-L3-CM-P1						
Penicillium decumbens	NR_111133.1	100%	*Penicillium decumbens*	MN396675	100%	2	FJII-L3-CM-PAB4	FKII-L3-CM-DRAB1					
Aspergillus pseudodeflectus	NR_135372.1	98%	*Aspergillus*	KJ567455	98%	7	FKI-L3-BK-P1	FKI-L3-BK-DRAB1	FKII-L2-CM-DR1	FKII-L2-CM-PAB1	FKII-L3-BK-PAB1	FKII-L3-BK-DRAB4	FKI-L3-BK-PAB1
Exophiala oligosperma	NR_111134.1	99%	*Exophiala oligosperma*	KP938216	100%	1	FKI-L8-BK-P1						
Ganoderma meredithae	NR_164435.1	91%	*Ganoderma zonatum*	MG654423	100%	1	FKI-L8-BK-PAB1						
Penicillium chrysogenum	NR_077145.1	100%	*Penicillium chrysogenum*	MK267450	100%	1	FKI-L3-BK-PAB3						
Cystobasidium benthicum	NR_171726.1	97%	*Cystobasidium benthicum*	FJ515210	98%	1	FKI-L6-BK-PAB1						
Barbatosphaeria neglecta	NR_132088.1	85%	*Ceratostomella pyrenaica*	MH865227	96%	1	FKII-L8-BK-P4						
Vagicola arundinis	NR_154541.1	92%	*Paraphoma fimeti*	MF494612	100%	1	FKII-L2-CM-DR2						
Acremonium citrinum	NR_154670.1	95%	Ascomycota	AF502849	98%	1	FKII-L8-BK-P5						
Cladosporium colombiae	NR_119729.1	100%	*Cladosporium colombiae*	MT804602	100%	1	FKII-L3-CM-PAB1						
Phaeophleospora eucalypticola	NR_145123.1	99%	*Phaeophleospora eucalypticola*	KX228267	99%	1	FKII-L3-CM-PAB3						
Talaromyces veerkampii	NR_153228.1	99%	*Talaromyces flavus*	MK734062	99%	3	FKII-L3-CM-P1	FKII-L2-CM-P1	FKII-L2-CM-DRAB3				
Alternaria alstroemeriae	NR_163686.1	100%	*Alternaria alternata*	MT279999	100%	2	FKII-L8-BK-P2B	FKII-L2-CM-DRAB1					
Cladosporium endophytica	NR_158360.1	100%	*Cladosporium halotolerans*	MH864391	100%	1	FKII-L2-CM-DR3						

Aspergillus costaricaensis	NR_103604.1	100%	*Aspergillus niger*	MW193054	100%	1	FKII-L6-BK-DRAB1		
Aspergillus caatingaensis	NR_172047.1	98%	*Aspergillus turcosus*	MN431383	100%	2	FKII-L3-BK-DRAB1	FKII-L3-BK-DR1	
Eutypa microasca	NR_153990.1	88%	*Eutypa*	KU204532	99%	1	FKII-L8-BK-P1		
Aureobasidium melanogenum	NR_159598.1	99%	*Aureobasidium pullulans*	MT936537	100%	3	FJII-L3-CM-P3	FKI-L6-BK-P2	FKI-L2-BK-P2
Talaromyces ruber	NR_111780.1	99%	*Talaromyces ruber*	LT558959	100%	1	FKI-L3-BK-DAB3		
Botryosphaeria agaves	NR_111792.1	82%	Fungi	KF800689	99%	1	FJII-L1-SW-P2		
Arthrocladium tropicale	NR_154724	85%	Fungi	KX515916	94%	3	FJII-L3-CM-DR1	FJI-L2-BK-P2	FJII-L6-SW-P1
Pseudopyrenochaeta terrestris	NR_160575.1	87%	*Pyrenochaeta*	KT581913	91%	1	FJII-L10-SW-DR1		
Vexilomyces palatinus	NR_165532.1	96%	Fungi	EU480201	87%	1	FKI-L1-BK-DR1		

, JII - JPL-SAF

KI, KII - KSC-PHSF

24hr enrichment with antibiotic

UV resistant to UV-C dose 1,000 J/m^2

Isolates with similarity lower than 97% to any known species - novel species
