# Supplementary material for: The Isolation and Characterization of Rare Mycobiome Associated With Spacecraft Assembly Cleanrooms
Source: Front Microbiol. 2022 Apr 26;13:777133. doi: 10.3389/fmicb.2022.777133 (PMC9087587; doi:10.3389/fmicb.2022.777133)
Supplement: Supplementary file 4 [file Table_4.PDF]

Supplementary Table 4. Viable ITS amplicon sequencing reads in JPL-SAF and KSC-PHSF cleanrooms

Phylum	Genus	JPL-1		JPL-2		KSC-1		KSC-2	
		No AB	AB	No AB	AB	No AB	AB	No AB	AB
Ascomycota	*Alternaria*				3,963		136		
	Aspergillus	1,824		108,847	14,616		9,511		20,897
	Candida			118	414	8,839	841		10,054
	Cladosporium		2,253	10,890	2,645		2,460	12,703	
	Cytospora				2,191				
	Dermateaceae		2,000						
	Didymella						20,592		61,133
	Helotiales				2,324				
	Hormonema		760						
	Knufia								10,795
	Mycosphaerella						10,042		
	Penicillium			425	8,846		6,051		
	Peniophora							816	
	Pyrenochaetopsis	8,395							
	Saccharomyces				1,982				
	Saccharomycetales				3,702				
	Stachybotrys						8,327		
	Taphrina	3,381							
	Trichocomaceae					1,468			
	Verrucocladosporium				2,265				
	Wallemia				3,565				
	Xylariales		672						
Basidiomycota	*Boletus*						7,300		
	Cyberlindnera	284					501		10,065
	Cystobasidium						242		10,086
	Erythrobasidium								26,604
	Fomitopsidaceae							1,306	
	Hannaella	2,284			1,176	1,422			13,786
	Hymenochaetopsis			7,010					
	Hyphodontia		1,551						
	Psathyrella						7,785		
	Sporobolomyces							1,183	
Unclassified	Unidentified	56,663	3,947	119	7,021	8,357	2,943		77,183
	Other (< 3%)	5,119	709	1,935	6,063		18,948		9,116
Total number of reads		77,950	11,892	129,344	60,773	20,086	95,679	16,008	249,719

* abundance below 3% of total reads from a given location with or without the antibiotic treatment
