# Supplementary material for: The Isolation and Characterization of Rare Mycobiome Associated With Spacecraft Assembly Cleanrooms
Source: Front Microbiol. 2022 Apr 26;13:777133. doi: 10.3389/fmicb.2022.777133 (PMC9087587; doi:10.3389/fmicb.2022.777133)
Supplement: Supplementary file 5 [file Table_5.PDF]

Supplementary Table 5. Viable fungal metagenome reads at JPL-SAF and KSC-PHSF cleanrooms

Species	JPL-1		JPL-2		KSC-1		KSC-2	
	no AB	AB	no AB	AB	no AB	AB	no AB	AB
Alternaria alternata	6	2	2	15		58	12	287
Ascochyta rabiei	8			4		646	1	227
Aspergillus calidoustus				6,578		20,778		
Aspergillus fumigatus				244		248		
Aspergillus sydowii		1		312	4	120		1
Aspergillus terreus				10		17	53	
Aspergillus udagawae				355	1	26		
Candida intermedia		5						
Candida tropicalis					92	1,265		
Cyberlindnera jadinii	14	12			133	10,790	89	198
Epicoccum nigrum	7		1	16		482		241
Fomitiporia mediterranea		3	2	5		3		
Gymnopus luxurians			2	13	28	1		2
Hortaea werneckii	1	2	1	1		13	16	159
Leptosphaeria maculans	6					12		7
Malassezia globosa	20	50			376	330		
Malassezia pachydermatis	2	2	26	88	5	18	38	7
Malassezia sympodialis	2	6	33	81	18	23	42	96
Penicillium chrysogenum						1,577		1
Penicillium rubens				3		7,936		
Pestalotiopsis fici			23	36	107	32		
Puccinia striiformis			2	24	10	3	43	7
Purpureocillium lilacinum	2		2	1		36,645		
Pyrenochaeta sp. DS3sAY3a	20			1		32		
Rhizophagus irregularis		3	8	44	5	12	17	7
Schizophyllum commune		5			1			
Serendipita indica					1	1		85
Serendipita vermifera								325
Talaromyces marneffei				18	25	52	2	2
Talaromyces stipitatus				11	31	18		1
Talaromyces verruculosus				1	24	17		
Tilletia caries			2	20	37	9		
Other (< 0.6%)*	56	25	205	1,413	278	7,095	631	1,048
Annotated reads	144	116	309	9,294	1,176	88,259	944	2,701
Total reads	913	349	55,980	38,963	3,511	246,132	4,946	13,322

* abundance below 0.6% of total reads from a given location with or without the antibiotic treatment
