# Supplementary material for: The Isolation and Characterization of Rare Mycobiome Associated With Spacecraft Assembly Cleanrooms
Source: Front Microbiol. 2022 Apr 26;13:777133. doi: 10.3389/fmicb.2022.777133 (PMC9087587; doi:10.3389/fmicb.2022.777133)
Supplement: Supplementary file 6 [file Data_Sheet_1.docx]

Supplementary Material

**Antibiotic treatment allows for the isolation and characterization of rare mycobiome associated with spacecraft assembly cleanrooms**

**Adriana Blachowicz^1¢^, Snehit Mhatre^1¢^, Nitin Kumar Singh^1^, Jason M. Wood^3^, Ceth W. Parker^1^, Cynthia Ly^1^, Daniel Butler^2^, Chrristopher E. Mason^2,3^, Kasthuri Venkateswaran^1*^**

*** Correspondence:** Dr. Kasthuri Venkateswaran (Venkat): kjvenkat@jpl.nasa.gov

# Supplementary Data

**Data set 1:** Summary of fungal populations identified in PMA treated and untreated samples assessed via amplicon sequencing

**Data set 2:** Summary of ITS amplicon reads present in PMA-treated samples and controls during each sampling event

**Data set 3:** Summary of fungal populations identified in PMA treated and untreated samples assessed via metagenome sequencing

**Data set 4:** Summary of metagenomic reads present in PMA-treated samples and controls during each sampling event

# Supplementary Figures and Tables

## Supplementary Figures

**
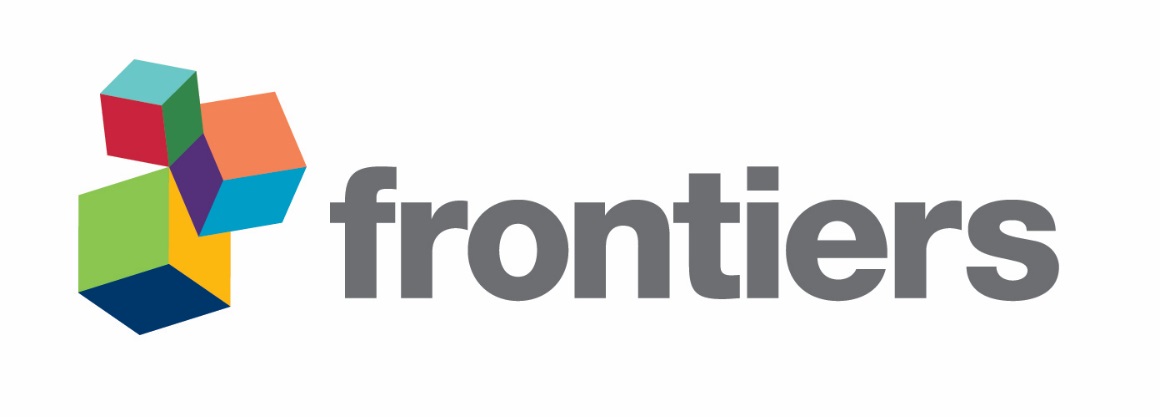
**

**Supplementary Figure 1.** The figure legends are required to have the same font as the main text, 12 point normal Times New Roman, single spaced. Please use a single paragraph for each legend and prepare the figures keeping in mind the PDF layout.

**Supplementary Figure 1. Total number of viable reads from JPL-SAF and KSC-PHSF. a)** Total number of identified OTUs via ITS amplicon sequencing. **b)** Total number of viable reads identified via ITS amplicon sequencing. **c)** Total number of metagenomic reads.

**Supplementary Figure 2. Schematic representation of sampling locations at JPL-SAF (a) and KSC-PHSF (b) cleanrooms. (a)** Locations sampled during the first event on 17 April, 2018 are marked in light orange and numbered L1-L10, while areas sampled during the second sampling on 25 September, 2018 are marked in light blue. Areas that were sampled during both sampling events included L1, L7, L9 and L10, remaining locations L11-L16 were adjusted for the second sampling due to the presence of critical hardware and ground support equipment in JPL-SAF. ClipperMop samples were collected during the second sampling only and are labeled as C1-C3. The graph is sectioned into artificial quadrants based on sample grouping and foot traffic. (**b)** Locations L1-L10 and C1-C3 are marked in green/purple rectangles, and were duplicated during both sampling events on 12 June, 2018 and 24 July, 2018, respectively. The graph is sectioned into artificial quadrants for sampling distribution, not based on foot traffic, as there was no ongoing assembly activity at the time of both samplings.

**Supplementary Figure 3. Schematic sample processing flow-chart.** All the steps and procedures followed to process the surface samples from both cleanrooms after each sampling event are depicted.

## Supplementary Tables

**Supplementary Table 1:** Cultivable fungal burden of JPL-SAF. A) Sampling on 04/17/2018 B) Sampling on 09/25/2018.

**Supplementary Table 2:** Cultivable fungal burden of KSC-PHSF. A) Sampling on 06/12/2018 B) Sampling on 07/24/2018.

**Supplementary Table 3:** ITS identification of isolates collected from JPL-SAF and KSC-PHSF cleanrooms

**Supplementary Table 4:** Viable ITS amplicon sequencing reads in JPL-SAF and KSC-PHSF cleanrooms

**Supplementary Table 5:** Viable fungal metagenome reads at JPL-SAF and KSC-PHSF cleanrooms
